# The impact of chronic pain on creative ideation: An examination of the underlying attention‐related psychophysiological mechanisms

**DOI:** 10.1002/ejp.2000

**Published:** 2022-07-13

**Authors:** Danièle Anne Gubler, Christian Rominger, Martin grosse Holtforth, Niklaus Egloff, Frank Frickmann, Benjamin Goetze, Michael Harnik, Konrad Streitberger, Stephan Zeiss, Stefan Johannes Troche

**Affiliations:** ^1^ Department of Psychology University of Bern Bern Switzerland; ^2^ Department of Psychology Universtiy of Graz Graz Austria; ^3^ Division of Psychosomatic Medicine, Department of Neurology Inselspital, Bern University Hospital, University of Bern Bern Switzerland; ^4^ Spital Aarberg Inselgruppe AG Aarberg Switzerland; ^5^ Department of Anaesthesiology and Pain Medicine, Inselspital Bern University Hospital, University of Bern Bern Switzerland

## Abstract

**Background:**

Attentional deficits in patients with chronic pain are common and well studied. Yet, few studies have examined the effects of chronic pain on more complex cognitive abilities that rely on well‐functioning attentional systems. With the current study, we aimed to investigate whether the impact of chronic pain on attention affects creative ideation as measured with an adaptation of the alternate uses task (AUT).

**Methods:**

Performance in the AUT was compared between 33 patients suffering from chronic pain and 33 healthy matched controls. While solving the task, EEG was recorded to measure the degree of internally directed attention assessed by means of task‐related power (TRP) changes.

**Results:**

The results revealed that patients with chronic pain generated less creative ideas than healthy controls. This lack of performance was accompanied by lower event‐related synchronization (ERS), especially in right parietal sites. Furthermore, these ERS differences explained one‐third of the inter‐group variance in AUT performance.

**Conclusions:**

These results suggest that performance decrements in creative ideation in patients with chronic pain may be at least partly attributable to attentional impairments associated with chronic pain.

**Significance:**

Chronic pain negatively affects attention and more complex cognitive abilities. However, the underlying psychophysiological mechanisms and the role of attention as a source of these impairments in more complex abilities are poorly understood. By analyzing task‐related power changes in the EEG, the role of internal attention in creative ideation could be determined, revealing the functional relationship between chronic pain, attention, and a more complex cognitive ability.

## 1 INTRODUCTION

The disruptive effect of chronic pain on attention has been observed in experimental tasks on different kinds of attention (Higgins et al., 2018; Moriarty et al., 2011) as well as in changes in attention‐related brain activity measured by the electroencephalogram (EEG; Pinheiro et al., 2016). However, the potential impact of chronic pain on more complex cognitive processes that rely on well‐functioning attentional systems is less well understood. Only in recent years, some behavioural studies provided evidence for the adverse effect of chronic pain on the performance of abstract thinking (Gunnarsson & Agerström, 2018), daily decision‐making (Attridge et al., 2019) and, to some extent, logical reasoning (Gunnarsson & Agerström, 2021). While these findings suggest that chronic pain may lead to a broader range of cognitive impairments, the underlying psychophysiological mechanisms and the role of attention as the driving source of these impairments have hardly been addressed.

A cognitive ability that is essential for everyday functioning is creative ideation. Creative ideation involves generating many different and creative (i.e. unusual) solutions to a given problem and is an integral process underlying a person's creative potential (Fink & Benedek, 2014; Runco & Acar, 2012). The role of attention in creative ideation has been studied extensively at the behavioural and psychophysiological levels, and internally directed attention (i.e. the allocation of attention to internal mental representations) seems to be particularly important for the production of creative ideas (Benedek, 2018).

According to Fink and Benedek (2014), internal attention during creative ideation is reflected in the activity of the upper alpha band (10–12 Hz) in the right posterior hemisphere. This reasoning is based on four key findings: 1. Alpha power, especially in right posterior brain areas, increases from a reference state to a state of creative ideation, also referred to as event‐related synchronization (ERS; Jausovec, 1997; Martindale & Hasenfus, 1978; Mölle et al., 1999; Pfurtscheller & da Silva, 1999). 2. This ERS increases with increasing creativity‐related task demands (Fink et al., 2007; Jauk et al., 2012; Mölle et al., 1999). 3. ERS is higher for more creative ideas compared to less creative ideas in the same individuals (Fink & Neubauer, 2006; Grabner et al., 2007; Schwab et al., 2014). 4. More creative individuals exhibit higher ERS than less creative individuals (Fink et al., 2009; Fink & Neubauer, 2008; Rominger et al., 2019).

With the present study, we applied an adaptation of the alternate uses task (AUT; Guilford, 1967) to measure creative ideation in patients with chronic pain and healthy matched controls. Given that creative ideation depends on attention (Benedek, 2018) and that attentional resources are impaired by chronic pain, our first hypothesis was that patients with chronic pain would give less creative responses in the AUT than healthy controls. To examine whether a potential difference in creative ideation performance between patients with chronic pain and healthy controls can be explained by impaired internal attention, we measured the power of the upper alpha band in the EEG during a reference phase and during a creative ideation phase. Our second hypothesis was that ERS at right posterior sites would be observed during creative ideation to reflect internal attention (Benedek, Schickel, et al., 2014) but be less pronounced in patients with chronic pain than in healthy controls. If the first two hypotheses were confirmed, we would further examine whether ERS differences explain AUT differences between the two groups to substantiate the assumption that attentional deficits underlie poorer performance on a creative ideation task in patients with chronic pain.

## 2 METHOD

### 2.1 Participants

The sample of patients with chronic pain consisted of 43 participants. Of these, four patients were excluded because they misunderstood the AUT, four were excluded because of poor EEG signal quality and two were excluded because they were identified as outliers (see Statistical Analysis). The final sample consisted of 33 patients^1^ (20 female, 13 male) ranging in age from 19 to 64 years (*M* = 42.4, *SD* = 14.1). Regarding educational level, two patients (6%) finished mandatory school, 22 (67%) finished an apprenticeship and 9 (27%) finished high school or higher education. Twenty‐nine patients (88%) reported being right‐handed. Patients were recruited at a Swiss tertiary psychosomatic university clinic and had been diagnosed with chronic pain disorders according to ICD‐10 (F45.41; R 52.1; R 52.2 chronic pain with somatic and psychological factors). Most patients had musculoskeletal pain (76%), followed by chronic headache (12%). Six percent of the patients did not give any information on the localization of their pain. For study inclusion, patients with chronic pain were required to speak German fluently and have a negative history of dementia, psychosis, brain concussion, neurological diseases, alcohol‐related disorder and intake of medications such as antihistamines, benzodiazepines or opioids. Medication intake was recorded by a physician. While four (12%) patients were prescribed concomitant antidepressants and anticonvulsants, thirteen (39%) patients were prescribed only antidepressants and two (6%) patients only anticonvulsants. Eleven (33%) patients were not taking any medication with a potentially sedating side effect. Twelve (36%) patients reported a pain duration from at least 1 to 5 years, six (18%) patients from 6 to 10 years and 15 (45%) patients more than 10 years.

As a control group, 33 healthy participants (20 female, 13 male) ranging in age from 20 to 64 years (*M* = 42.7, *SD* = 14.3) were recruited through (social) media advertisements and personal contacts. Twenty‐two (67%) of the participants finished an apprenticeship, and 11 (33%) finished high school or higher education. Twenty‐six (79%) of the participants reported being right‐handed. Inclusion and exclusion criteria were the same as for patients with chronic pain. Healthy participants were recruited to match the patients' gender, age, educational level, and handedness.

All participants were instructed not to drink alcohol, smoke cigarettes, drink caffeinated beverages or eat a large meal 1 h before the study. Since patients with chronic pain were stationed at a university hospital with a tight schedule, the experiment took place later in the afternoon. The healthy control subjects were therefore also tested later in the afternoon. All participants were informed about the study protocol and signed informed consent before testing. The study protocol is in accordance with the Declaration of Helsinki and was approved by the Ethics Committee of the Canton of Bern, Switzerland (project ID 2019‐01552).

### 2.2 Instruments

Actual and average pain intensity was measured on a visual analogue scale (VAS; Bijur et al., 2001). Subjects indicated the degree of (a) ‘actual pain’ and (b) ‘average pain during the past 24 hours’ on a horizontal VAS ranging from ‘no pain’ (0) to ‘worst pain imaginable’ (10). Furthermore, participants were asked to indicate their average sleep duration with the question: ‘How many hours of sleep did you get at night on average during the past month?’ Handedness was assessed by the 13 items of the Edinburgh Handedness Inventory (Oldfield, 1971).

To assess depression and anxiety, the Hospital Anxiety and Depression Scale was used (HADS; Zigmond & Snaith, 1983). The HADS consisted of seven items per scale measuring the extent of anxiety and depression. Bjelland et al. (2002) reported good internal consistencies (anxiety α = 0.83, depression α = 0.82) and high convergent validity with other measures of anxiety and depression. Mean scores across the seven items for each scale were computed to assess the extent of anxiety and depression.

Intelligence was assessed with the mini‐q (Baudson & Preckel, 2015). Subjects had to judge within 3 min on 64 different items whether a sentence describing a visually depicted symbol constellation was correct or incorrect. The mini‐q had high split‐half reliability, *r*_tt_ = 0.98, and high convergent validity with other measures of intelligence with correlations ranging from *r* = 0.37 to *r* = 0.73 (Baudson & Preckel, 2015).

### 2.3 Alternate uses of task

**FIGURE 1:**
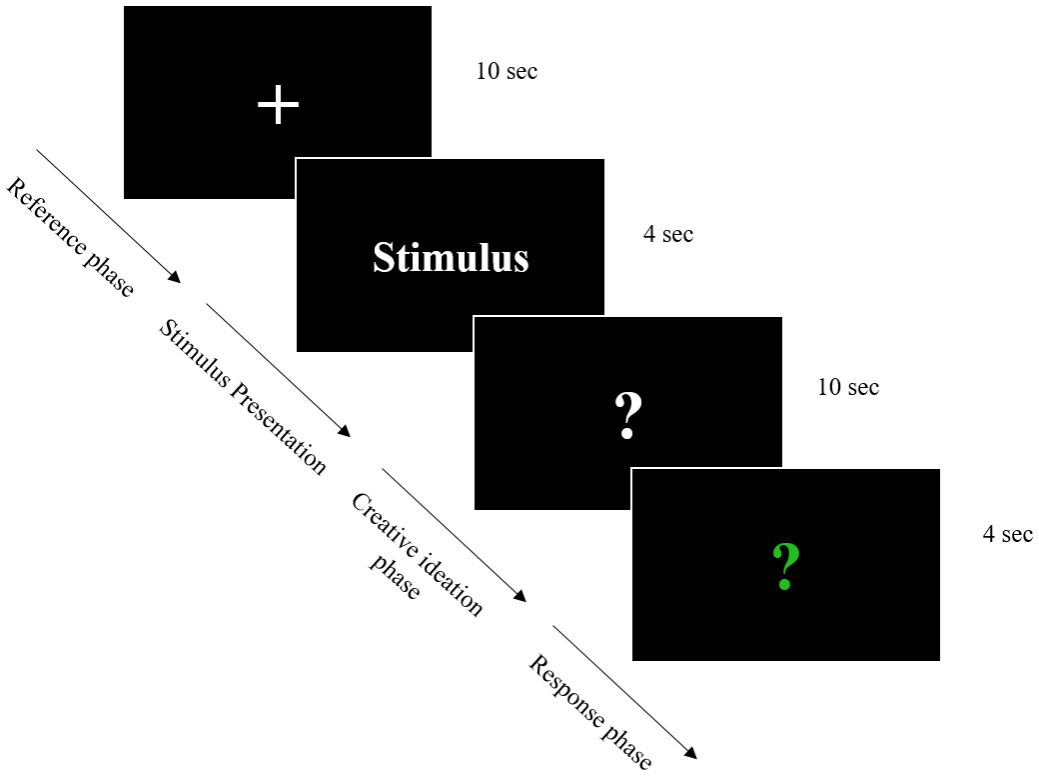
Procedure of a trial of the alternate uses task (AUT). Each trial started with a white fixation cross (reference phase) that was displayed on the screen for 10 s, followed by one of 20 different stimuli, to which test subjects had to generate the most original idea possible within 10 s (creative ideation phase). As soon as the question mark changed from white to green, the best idea could be pronounced. Afterward, the next trial followed (procedure and figure adapted from Schwab et al., 2014).

Creative ideation was measured with the AUT (Guilford, 1967) as adapted by Schwab et al. (2014). The AUT is a well‐validated verbal measure of creative ideation that has been previously used in a large number of neuroscientific studies on creativity (Fink et al., 2007; Rominger et al., 2019; Schwab et al., 2014; Wu et al., 2015). The role of internal attention in creative ideation as reflected in alpha ERS is probably best understood for this creativity test. As depicted in Figure 1, the AUT was presented via a computer screen. Participants were instructed that they would be presented with an everyday object with each trial and asked to find the most creative use possible. Instructions emphasized to generate a single use for that particular object that was as creative as possible but at the same time useful (e.g. umbrella as a sword, fork as a brush or brick as a beehive). Each of the 20 trials started with a white cross on the black screen (10 s; reference phase) followed by a 4‐s presentation of a word naming an ordinary everyday object (e.g. umbrella, fork, brick). Then, a white questionnaire was presented on the screen for 10 s before it turned into a green questionnaire. The onset of the white questionnaire indicated the beginning of the creative ideation phase. During the presentation of the green questionnaire, participants were asked to express their idea aloud verbally. Instructions emphasized that no response should be given as long as the question mark was white. The test administrator recorded the oral responses. In order to check whether the task was understood correctly, there were two practice trials before the actual task. After these practice trials, open questions could be clarified and the subjects could be briefed again if something was not understood correctly.

Four well‐instructed raters (two Ph.D. students and two research assistants) independently evaluated the creativity of the ideas. Items were rated by each rater in turn. For this purpose, all responses per item were listed in a separate Excel sheet. Responses were sorted alphabetically to facilitate how often multiple participants generated the same idea. In the first step, raters were supposed to check whether the idea met the criterion of usefulness/effectiveness (if not, it was rated as non‐creative—irrespective of its uniqueness/originality), and in the second step, raters were supposed to consider the uniqueness/originality of the idea. Raters were then instructed to give one rating per response. Thus, the ratings were based on the effectiveness as well as the uniqueness/originality of the response, which are the two essential criteria for a creative idea (Diedrich et al., 2015; Runco & Jaeger, 2012). Creativity ratings could be made on a 4‐point Likert scale ranging from not creative or not useful (1), useful but an ordinary idea/not really creative (2), useful and creative idea (3), to useful and very creative/an idea named by only a few participants (4). For example, an answer for the item fork as cutlery was rated with 1 point, a brush with 2 points, a screwdriver with 3 points and a power line with 4 points. The intra‐class correlation coefficient (ICC) showed good inter‐rater reliability for the creativity ratings, ICC (2,k) = 0.87. The four ratings were averaged for each idea and a creativity score was computed for each participant as the average score across the 20 trials. Raters did not know whether participants were patients or healthy controls.

### 2.4 Electroencephalogram recording and analysis

Brain activity was monitored using a mobile dry‐electrode EEG system (DSI 24) and the corresponding DSI‐STREAMER recording software. EEG was synchronized with the AUT automatically by sending triggers from the presentation computer to the EEG recording software during the EEG recording. According to the international 10–20 system, 21 electrodes (Fp1, Fp2, Fz, F3, F4, F7, F8, Cz, C3, C4, T7, T8, Pz, P3, P4, P7, P8, O1, O2, A1, A2) were arranged. In addition, an electrooculogram (EOG) was installed to measure eye movements. For this purpose, two horizontal electrodes were placed to the left and right of the eyes (horizontal EOG), while Fp2 and an electrode on the infraorbital ridge of the right eye were used to measure vertical EOG. During recording, Pz served as a reference electrode, which was re‐referenced to electrodes on the earlobes (A1 + A2) via DSI‐STREAMER recording software before exporting the data.

EEG and EOG were recorded at a rate of 300 Hz and resampled to 256 Hz using the software BrainVision Analyser 2.2. The continuous signal was further filtered offline (0.1–30 Hz). With Gratton and Coles’ (1989) ocular correction procedure, data were corrected for eye movements using the horizontal and vertical EOG and then visually inspected for movement artefacts, eye blinks and muscle tension to be removed from the EEG. Due to poor signal quality in electrodes F7/F8 (pulse artefacts) or T7/T8 (muscle tension), in approximately one‐quarter of the subjects (in eight patients with pain and in nine healthy controls), individual channels had to be replaced by applying interpolation by spherical splines (similar approach see Jia et al., 2021). In the next step, the EEG signal was divided into 9‐s segments of the reference phase (0.5 s after the onset of the white cross to 0.5 before the onset of the item) and the creative ideation phase (0.5 s after the onset of the white question mark to 0.5 before the onset of the green question mark). These 9‐s segments were further segmented into equal‐sized 1‐s segments, each with a 0.5‐s overlap (50%). Each 1‐s segment was then visually inspected to exclude potential segments with artefacts that had not been previously excluded. Artefact‐free segments were then submitted to Fast Fourier Transformation (FFT) using a Hanning window for power estimates. The results were then averaged across all segments separately for the reference phase and the creative ideation phase per participant. Power scores for the frequency range from 10 to 12 Hz were extracted from the resulting FFT analysis.

Brain activity during the performance of the AUT was measured by means of TRP changes in the EEG (Pfurtscheller & da Silva, 1999). Task‐related power at an electrode [i] was obtained by subtracting log‐transformed power during the pre‐stimulus reference interval (Pow_i, reference_) from log‐transformed power during the creative ideation interval (Pow_i, creative ideation_) according to the formula: TRP = log(Pow_i, creative idation_) − log(Pow_i, reference_) (Fink et al., 2018; Jauk et al., 2012; Schwab et al., 2014). While negative values indicated power decreases from the reference to the activation interval (i.e. event‐related desynchronization, ERD), positive values indicated power increases (i.e. ERS) from the reference to the activation interval.

### 2.5 Statistical analysis

Previous studies examining performance differences in cognitive tasks between patients with chronic pain and healthy individuals have found medium to large effect sizes for tasks measuring attention, executive functions and working memory (see meta‐analysis by Wu et al., 2018). We, therefore, expected a moderate to large effect size (*f* = 0.40) for the difference in AUT scores between patients with chronic pain and healthy controls. Provided with an α error probability of *α* = 0.05 and statistical power of 1 − *β* = 0.85, the software G*Power (Faul et al., 2007) computed a minimum of 30 individuals per group to obtain significant results for the estimated effect with sufficient statistical power.

All analyses (except for the power analysis) were conducted using the statistical software RStudio version 1.4.1103. To test for differences in AUT scores between patients with chronic pain and healthy controls, a one‐way analysis of variance (ANOVA) with the between‐subjects factor ‘Group’ (patients vs. controls) and the dependent variable AUT score was conducted. For the analysis of TRP, a three‐way mixed‐model ANOVA was computed with one between‐subjects factor ‘Group’ (patients vs. controls), one within‐subjects factor ‘Position’ (eight electrode positions in each hemisphere) and one within‐subjects factor ‘Hemisphere’ (left vs. right). Post‐hoc pairwise comparisons were performed using Bonferroni‐Holm correction. Potentially significant TRP differences between patients with chronic pain and healthy controls were included as covariates in an ANCOVA with Group as the independent variable and AUT score as the dependent variable to test the impact of TRP differences on potential AUT differences between patients with chronic pain and healthy controls. Anxiety, depression and sleep duration were also considered potential covariates to rule out that the effects found were attributable to these potential confounding variables. To compare the effect of Group between ANOVA and ANCOVA, partial $\eta_{p}^{2}$ was calculated, respectively, and compared afterwards.

Furthermore, ANOVAs or correlation analyses were used to examine how the influence of antidepressants and pain intensity affected AUT scores and TRP values within the group of patients with chronic pain.

Before conducting the ANOVAs, several assumptions regarding the absence of outliers, normality, homogeneity of variance and sphericity were checked (Tabachnik & Fidell, 2019). Participants with values of more than three standard deviations above or below the mean were identified as outliers and removed from further analysis resulting in the two outliers in the group of patients with chronic pain mentioned above in the description of participants. If Mauchly's test of sphericity was significant, Greenhouse–Geisser correction was applied. For ANCOVA, potential covariates were first correlated with the dependent variable to determine whether a significant relationship existed. If a correlation was significant and regression slopes were considered homogeneous, variables were included as covariates in the analyses. Data and the analysis script are publicly available at the Open Science Framework and can be accessed at osf.io/5g497.

## 3 RESULTS

Descriptive statistics of pain scores, depression, anxiety, sleep duration and intelligence scores are presented in Table 1. Also given in Table 1 are the test statistics to compare patients with chronic pain and healthy controls regarding these variables. Patients with chronic pain reported significantly more actual pain and average pain than healthy controls within the last 24 h. In patients with chronic pain, the current pain scores ranged from 1 to 8, and the scores for average pain within the last 24 h from 2 to 10. In contrast, 30 healthy controls did not suffer from pain at all, while three individuals reported current pain scores of 1 or 2. Moreover, patients with chronic pain scored significantly higher on the depression and anxiety scale than healthy controls. However, both groups did not significantly differ in average sleep duration during the past month nor in intelligence scores.

**TABLE 1:** Mean (M), standard deviation (SD), Welch's *t* test and Cohen's d for mini‐q scores, sleep duration, anxiety and depression scores as well as actual pain and average pain in the last 24 h for 33 patients with chronic pain and 33 healthy controls, respectively

	Patients with chronic pain		Healthy controls		Welch's *t*	Cohen's *d*
	*M*	*SD*	*M*	*SD*		
Mini‐q scores	21.85	9.35	25.64	7.47	−1.817	−0.45
Average sleep duration (last month)	6.62	1.22	6.97	1.05	−1.247	−0.31
HADS Depression Score (1–4)	2.04	0.62	1.36	0.29	5.682***	1.40
HADS Anxiety Score (1–4)	2.10	0.52	1.63	0.37	4.200***	1.03
Actual Pain Score (0–10)	4.67	1.88	0.12	0.42	13.549***	3.34
Average Pain Score (last 24 hr, 0–10)	5.46	2.05	0.03	0.17	15.162***	3.73

*Note*: Abbreviation: HADS, Hospitality Anxiety and Depression Scale.

*** *p* < 0.001.

The one‐way ANOVA on the AUT scores of patients with chronic pain and healthy controls yielded a significant main effect Group, *F*(1,64) = 6.369, *p* = 0.014, $\eta_{p}^{2}$ = 0.091. AUT scores of patients with chronic pain were significantly lower (*M =* 2.096, *SD* = 0.23) than the AUT scores of healthy controls (*M =* 2.212, *SD* = 0.13). Although both groups had an average score between ordinary ideas and creative ideas, the average responses of patients with chronic pain were significantly closer to the anchor of ordinary ideas (i.e. ‘2’) than healthy controls' responses. As can be taken from Table 2, neither in patients with chronic pain nor in healthy controls depression, anxiety and sleep duration significantly correlated with AUT scores. These variables could therefore be excluded as potential covariates.

**TABLE 2:** Zero‐order correlation for AUT score, depression, anxiety, sleep duration and pain scores separated for 33 patients with chronic pain (below the diagonal) and 33 healthy controls (above the diagonal)

	1.	2.	3.	4.	5.	6.
1. AUT Score		0.05	−0.12	−0.10	0.23	0.00
2. HADS Depression Score	−0.07		0.44*	−0.26	0.11	−0.22
3. HADS Anxiety Score	−0.07	0.68***		−0.05	0.16	−0.03
4. Average Sleep duration	0.30	−0.19	−0.04		0.04	0.09
5. Actual Pain Score	−0.57***	0.27	0.16	−0.38*		0.38*
6. Average Pain Score (last 24 h)	−0.43*	0.13	0.15	−0.24	0.75***	

*Note*: Abbreviation: HADS, Hospitality Anxiety and Depression Scale.

* *p* < 0.05.

*** *p* < 0.001.

In patients with chronic pain, actual and average pain scores were significantly negatively related to AUT scores with moderate to strong effect sizes (see Table 2). The higher the patient‐reported pain level, the worse their creative ideation performance. In addition, AUT scores of patients taking antidepressants were compared to AUT scores of patients not taking antidepressants. Although AUT scores of patients taking antidepressants were smaller (*M =* 2.029, *SD* = 0.27) than AUT scores of patients not taking antidepressants (*M =* 2.168, *SD* = 0.16), these scores did not differ significantly, *F*(1,31) = 3.212, *p* = 0.083, $\eta_{p}^{2}$ = 0.094. However, it should be noted that there was probably not enough statistical power to find a significant effect due to the small sample size. Since it can be assumed that patients suffering from more severe pain are more likely to take antidepressants, we additionally considered the results with acute pain scores as a covariate. When considering pain severity as a covariate, the difference in AUT between patients taking antidepressants and patients not taking antidepressants was further reduced, *F*(1,30) = 1.625, *p* = 0.212, $\eta_{p}^{2}$ = 0.051, while pain severity was a significant predictor of AUT scores, *F*(1,30) = 12.778, *p* = 0.001, $\eta_{p}^{2}$ = 0.229. This covariation of antidepressants and acute pain suggests that the present study is not suitable for systematically investigating the influence of antidepressants.

**FIGURE 2:**
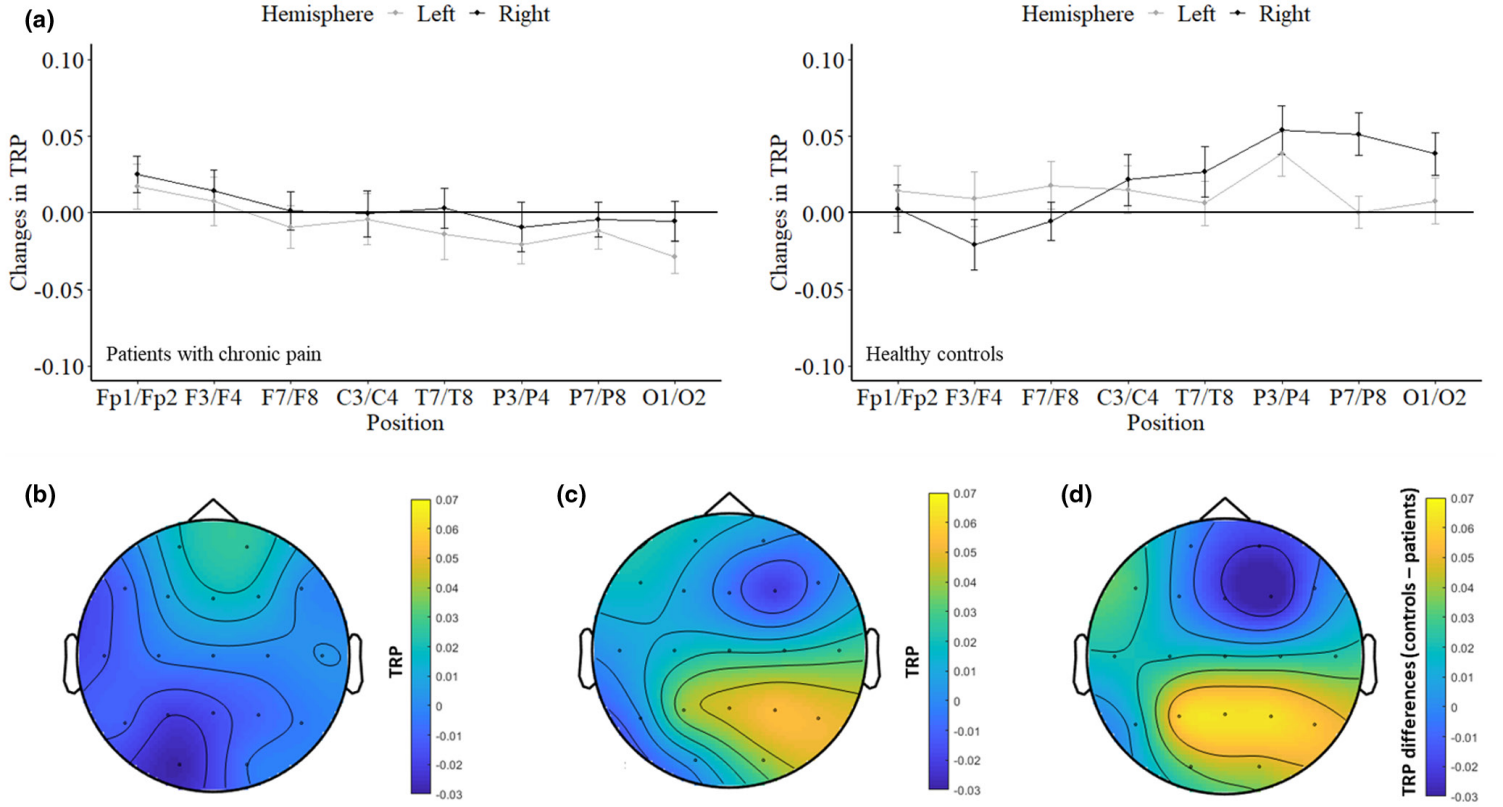
Means and standard errors of task‐related alpha power changes (10–12 Hz) during creative ideation between patients with chronic pain and healthy controls for eight cortical electrode sites of the right vs. the left hemisphere (a). TRP in patients with chronic pain (b), TRP in healthy controls (c), and TRP differences between healthy controls and patients with chronic pain (d).

Differences in TRP scores during creative ideation between patients with chronic pain and healthy controls were examined by means of a 2 (Group) × 8 (Position) × 2 (Hemisphere) mixed ANOVA with TRP values as the dependent variable. ANOVA yielded no main effect Group, *F*(1,64) = 2.337, *p* = 0.131, $\eta_{p}^{2}$ = 0.035, of Position, *F*(3.88,248.58) = 0.671, *p* = 0.609, $\eta_{p}^{2}$ = 0.010 and Hemisphere, *F*(1,64) = 2.740, *p* = 0.103, $\eta_{p}^{2}$ = 0.041, and no significant two‐way interaction Group × Hemisphere, *F*(1,64) = 0.099, *p* = 0.754, $\eta_{p}^{2}$ = 0.002. However, there were significant two‐way interactions Group × Position, *F*(3.88,248.58) = 3.880, *p* = 0.005, $\eta_{p}^{2}$ = 0.057, as well as “Position” × “Hemisphere”, *F*(5.20,332.82) = 3.133, *p* = 0.008, $\eta_{p}^{2}$ = 0.047. Furthermore, the three‐way interaction Group × Position × Hemisphere was statistically significant, *F*(5.20,332.82) = 2.274, *p* = 0.045, $\eta_{p}^{2}$ = 0.034, which is depicted in Panel A of Figure 2.

**FIGURE 3:**
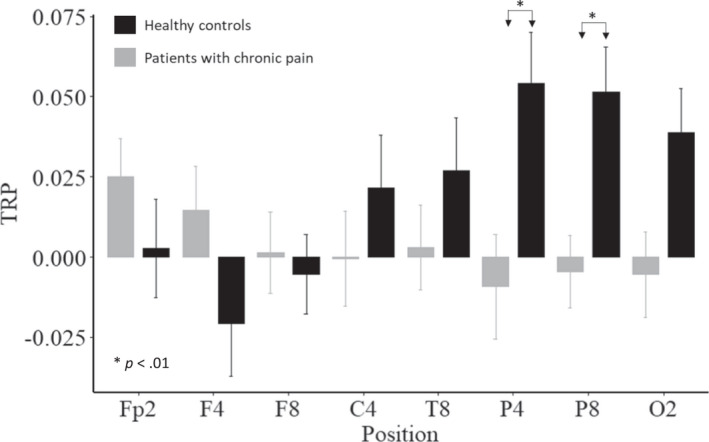
Means and standard errors of task‐related power changes (10–12 Hz) during creative ideation in the right hemisphere between patients with chronic pain and healthy controls.

To better understand the three‐way interaction, we considered the right and left hemispheres separately. In the left hemisphere, neither the main effects Group, *F*(1,64) = 2.109, *p* = 0.151, $\eta_{p}^{2}$ = 0.032, and Position, *F*(4.49,287.57) = 1.290, *p* = 0.271, $\eta_{p}^{2}$ = 0.020, nor the two‐way interaction Group × Position, *F*(4.49,287.57) = 1.653, *p* = 0.153, $\eta_{p}^{2}$ = 0.025, reached statistical significance. For the right hemisphere, the main effects Group, *F*(1,64) = 1.843, *p* = 0.179, $\eta_{p}^{2}$ = 0.028, and Position, *F*(4.08,260.99) = 1.497, *p* = 0.203, $\eta_{p}^{2}$ = 0.023, were also not significant. However, there was a significant two‐way interaction Group × Position, *F*(4.08,260.99) = 4.988, *p* < 0.001, $\eta_{p}^{2}$ = 0.072, in the right hemisphere. Bonferroni‐Holm corrected post‐hoc *t* tests for group mean differences yielded statistical significance at the parietal electrode sites P4, *t*(64) = −2.781, *p* = 0.007, Cohen's *d* = −0.685, and P8, *t*(64) = −3.102, *p* = 0.003, Cohen's *d* = −0.764 (see Figure 3). Patients with chronic pain had significantly lowered TRP values (less ERS) in P4 (*M* = −0.009, *SD* = 0.09) and in P8 (*M* = −0.004, *SD* = 0.07) than healthy controls, who showed the expected alpha power increases (P4: *M* = 0.054, *SD* = 0.09; P8: *M* = 0.051, *SD* = 0.08, see Figure 3). Although there was a further large difference at the occipital electrode site O2, *t*(64) = −2.300, *p* = 0.025, Cohen's *d* = −0.566, this was no longer significant after Bonferroni‐Holm alpha adjustment, *α* = 0.008 (0.05/6). For all other electrode sites, the differences between patients and healthy controls were not significant, all *t*s ≤ 1.659, *p*s ≥ 0.102, Cohen's *d*s ≤ 0.408.

TRP values at P4 and P8, where patients and healthy controls significantly differed, were averaged for each participant for further analyses. These TRP values were then correlated with anxiety, depression and sleep duration to examine the influence of possible covariates. As results revealed that there were significant relations of TRP values with anxiety, *r =* −0.291, *p =* 0.018, and depression scores, *r =* −0.245, *p =* 0.047, but not with sleep duration, *r =* 0.079, *p =* 0.530, anxiety and depression were considered as covariates. When taking anxiety into account as a covariate, the effect of Group remained a significant predictor of TRP values, *F*(1,63) = 5.444, *p* = 0.023, $\eta_{p}^{2}$ = 0.080, while the effect of anxiety did not reach statistical significance, *F*(1,63) = 1.303, *p* = 0.258, $\eta_{p}^{2}$ = 0.020. When taking depression into account as a covariate, Group further remained a significant predictor of TRP values, *F*(1,63) = 5.930, *p* = 0.018, $\eta_{p}^{2}$ = 0.086, while the covariate depression did not reach statistical significance, *F*(1,63) = 0.093, *p* = 0.761, $\eta_{p}^{2}$ = 0.001.

We further examined the averaged TRP values in relation to pain severity and antidepressants within the group of patients with chronic pain. Results revealed no significant relation between TRP values and acute pain, *r =* −0.058, *p* = 0.749, and between TRP values and average pain, *r* = 0.002, *p* = 0.991. Moreover, TRP values of patients taking antidepressants (*M =* −0.015, *SD* = 0.04) and patients not taking antidepressants (*M =* 0.001, *SD* = 0.09) did not significantly differ, *F*(1,31) = 0.411, *p* = 0.526, $\eta_{p}^{2}$ = 0.013.

As we observed significant differences in both AUT scores and TRP changes at parietal sites between patients with chronic pain and healthy subjects, we tested in a final step whether TRP differences at parietal electrode sites could explain the group difference in AUT scores. As this mean TRP value correlated significantly with AUT scores, *r* = 0.257, *p* = 0.037, we considered it as a covariate in the one‐way ANOVA on AUT differences between patients with chronic pain and healthy controls calculated in the first step. The resulting ANCOVA revealed that the difference in AUT scores between patients with chronic pain and healthy controls was no longer significant when controlled for mean parietal TRP values, *F*(1,63) = 3.463, *p* = 0.067, $\eta_{p}^{2}$ = 0.052. The effect of the covariate mean parietal TRP value did not reach statistical significance as well, *F*(1,63) = 1.738, *p* = 0.192, $\eta_{p}^{2}$ = 0.027. Effect size η_p_^2^ of the main effect “Group” decreased from $\eta_{p}^{2}$ = 0.091 in the ANOVA to $\eta_{p}^{2}$ = 0.052 in the ANCOVA, suggesting that about one‐third of the inter‐group variance in the AUT scores could be explained by inter‐group variance in TRP values.

Given that a number of recent findings pointed to enhanced resting‐state alpha activity for broader aspects of chronic pain (Pinheiro et al., 2016), we were interested in a final step to determine whether, in addition to the TRP differences, there were differences in alpha power during the reference phase and the activation phase between patients with chronic pain and healthy controls. For this reason, we examined the log‐transformed alpha power during the pre‐stimulus reference interval, on the one hand, and the log‐transformed alpha power during the creative ideation interval, on the other hand, for differences between patients with chronic pain and healthy controls. For this purpose, we conducted unpaired *t* tests with the independent variable Group (patients with chronic pain vs. healthy controls) and the dependent variable averaged alpha power at the electrode sites P4 and P8, separately for the reference phase and for the creative ideation phase. Patients with chronic pain showed significantly lower alpha power during the reference phase (*M* = 0.175, *SD* = 0.31) than healthy controls (*M* = 0.421, *SD* = 0.41), *t*(64) = −2.776, *p* = 0.007, Cohen's *d* = −0.683. The same pattern of results was found for the activation phase with patients having lower alpha power (*M* = 0.162, *SD* = 0.30) than healthy controls (*M* = 0.474, *SD* = 0.43), *t*(64) = −3.403, *p* = 0.001, Cohen's *d* = −0.838.

## 4 DISCUSSION

Attentional deficits are common in patients with chronic pain, and several studies have shown that chronic pain impairs performance on various attentional tasks (Higgins et al., 2018; Moriarty et al., 2011). Yet, few studies have examined the effects of chronic pain on the performance of more complex cognitive abilities that rely on well‐functioning attentional systems. With the present study, we examined whether chronic pain impairs performance in a creative ideation task. We observed that patients with chronic pain generated less creative ideas than healthy controls. Moreover, the greater the patients' reported pain, the less creative were their generated ideas. EEG recordings further revealed significantly less ERS (lower TRP values) during the creative ideation phase in patients with chronic pain than in healthy subjects, especially in parietal sites of the right hemisphere. Since lower ERS during creative ideation has been associated with lower internal attention (Benedek, Schickel, et al., 2014), these results may suggest that these processes were impaired in patients with chronic pain. These ERS differences explained about one‐third of the observed differences in creative ideation between patients with chronic pain and healthy controls.

Our findings are consistent with previous studies that demonstrated the detrimental effects of chronic pain on the performance of more complex cognitive abilities. In addition to the previously established adverse impact of chronic pain on abstractive thinking (Gunnarsson & Agerström, 2018) and decision‐making (Attridge et al., 2019), creative ideation also appears to be impaired by chronic pain. Specifically, higher severity of chronic pain was associated with lower performance, which resembled the previously reported effect of pain intensity on logical reasoning (Gunnarsson & Agerström, 2021). Since other potential confounding variables such as anxiety, depression and sleep duration did not significantly affect creative ideation, the results suggest that the impairments in creative ideation were most likely caused by pain and not by these other potentially influential variables. Nevertheless, it should be noted that the group differences in the AUT scores and the accompanying brain activity may still be explained by further confounding variables not considered in the present study. For instance, regardless of sleep duration, the overall level of fatigue may have been more present in chronic pain patients who were hospitalized and had a rigorous daily schedule than in healthy individuals. Since fatigue often accompanies chronic pain (Van Damme et al., 2018) and negatively affects cognitive performance (Lock et al., 2018), it could also affect the effects of chronic pain on performance. In addition, medications could have further influenced the results. Although we did not find significant differences between patients taking antidepressants and patients not taking antidepressants, their impact would need to be systematically investigated with a larger sample.

Regardless of these possible confounding variables, impaired creative ideation in patients with chronic pain can have severe consequences for their daily lives, as many activities require and benefit from creative thinking (Beaty, 2015; Boccia et al., 2015; Fink et al., 2019; Gajda et al., 2017; Ismail et al., 2019; Williams, 2004). As creative ideation generally facilitates dealing with difficult situations by enabling individuals to use different strategies (Falat, 2000; Schmidt, 2006), it might also be helpful in coping with pain (Schmidt, 2006). In addition, creative activities in themselves can have a curative and protective impact on psychological well‐being, as they promote relaxation, provide opportunities for self‐expression and relieve stress (Leckey, 2011).

Our findings provide further insight into the underlying mechanisms of creative ideation impairments in patients with chronic pain. Benedek, Schickel et al. (2014) have linked higher ERS in the upper alpha band at more parietal sites to increased internal attention. In order to develop creative ideas, associative elements stored in memory must be retrieved and combined (Benedek et al., 2012). No additional external information processing is required to carry out these processes (Benedek, 2018). Thus, the more attention can be allocated to these internal mental processes while shielding task‐irrelevant sensory input, the easier it is to generate more complex and creative mental representations, ultimately leading to more creative ideas (Benedek, 2018; Fink & Benedek, 2014). Increased ERS in more parietal areas in our healthy control subjects, therefore, suggests that they were able to direct their attention to internal mental processes during creative ideation. However, no such ERS from a resting state to a state of creative ideation was observed in patients with chronic pain. Since other explanatory variables could be excluded, chronic pain represents the most likely explanation for the absence of this increase, although it should be noted that chronic pain intensity was not directly associated with ERS in patients with chronic pain. This result is consistent with our hypothesis that chronic pain absorbs parts of the attention needed for creative ideation.

According to the fallacy of reverse inference pointed out by Poldrack (2006), a particular brain activation pattern during a cognitive task can be triggered by many different cognitive processes. Accordingly, inferring the involvement of a cognitive process from the activation of a brain region can be problematic (Hutzler, 2014). Applied to our results, we can conclude that creative ideation is accompanied by different activation patterns in the right parietal regions in patients with chronic pain compared to healthy controls. However, we cannot claim that these ERS differences are causally responsible for the group differences in performance in creative ideation. Therefore, to establish a functional relationship between chronic pain, internal attention and creative ideation, we used an ANCOVA to examine whether ERS differences could explain group differences in AUT. Indeed, ERS differences explained part of the performance differences in the AUT between patients with chronic pain and healthy controls. This finding suggests that impaired internal attention likely contributes to the deficits in creative ideation in patients with chronic pain. This aligns with Eccleston and Crombez's (1999) proposal that (chronic) pain leads to performance decrements because it drains limited attentional resources that are less available for other tasks. Given that attentional deficits could potentially translate into a broader range of other cognitive abilities, these findings may also help understand why people with chronic pain suffer from a wide range of cognitive impairments (Higgins et al., 2018; Moriarty et al., 2011).

However, ERS differences explained the group differences in creative ideas only partially. One explanation could be provided by the finding that patients with chronic pain not only differed in TRP values from healthy controls but also showed significantly different alpha power during both the reference and creative ideation phases. Several studies have demonstrated altered EEG activity in patients with chronic pain at a resting state (for a review see Pinheiro et al., 2016). Regarding the alpha band, an increase in alpha power at rest has been observed in patients suffering from chronic pain (Meneses et al., 2016; Sarnthein et al., 2006; van den Broeke et al., 2013). Alpha power is known to vary as a function of arousal and attentional demands (Hanslmayr et al., 2011; Klimesch et al., 1993). Surprisingly, compared to the above‐mentioned findings, alpha power in our sample was significantly lower in patients with chronic pain compared to healthy controls. However, in contrast to the studies mentioned above, in which alpha power was measured over a longer resting phase, alpha power in this study was measured over shorter reference periods between the AUT items. These reference periods have been established to investigate ERS during creative ideation in the AUT but might not adequately reflect a *resting* state. This might explain why in the present study alpha power was lower and not larger in patients with chronic pain compared to healthy controls. However, whether this conclusion is tenable would need to be investigated in further studies. Nevertheless, the altered alpha power found in patients with chronic pain could be indicative of functional, attention‐related changes in the brain.

Other explanations that the effect of ERS differences only partially explained the group differences might arise from studies that have shown structural changes in the brains of patients with chronic pain (Baliki et al., 2011; Mazza et al., 2018) that EEG cannot reveal. For example, grey matter volume has been observed to decrease in different brain regions in patients with chronic pain (May, 2008; Rodriguez‐Raecke et al., 2013). This structural change has further been linked to performance decrements in memory and executive functions (Lee et al., 2015; Luerding et al., 2008; Mazza et al., 2018). In addition to accessing knowledge and retrieving information, executive functions also facilitate creative ideation in several ways (Benedek, Jauk, et al., 2014; Gilhooly et al., 2007). In order to complete a creative ideation task, task instructions must be remembered and mentally processed, which involves adequate working memory performance (De Dreu et al., 2012; Lee & Therriault, 2013). Cognitive flexibility further facilitates breaking out of habitual thought patterns (Nusbaum & Silvia, 2011), and inhibitory control may be necessary to overcome interference by obvious (but not creative) ideas (Cassotti et al., 2016). Structural changes caused by chronic pain could thus also affect performance on creative ideation; however, this reasoning would require further investigations.

In sum, the present study provided evidence that attentional deficits in patients with chronic pain translate into a broader spectrum of cognitive impairments. More specifically, chronic pain impairs internal attention and, thereby, impedes creative ideation. The simultaneous use of behavioural and attention‐related psychophysiological measures allowed us to elucidate the functional relationship between attentional disruption by chronic pain and its impact on creative ideation. As attention also plays a crucial role in many other cognitive abilities, it would be compelling for further studies to explore whether similar mechanisms of the disruptive effects of chronic pain on attention can be identified that lead to these deficits. If such further functional relationships were uncovered, targeted attention‐related interventions could be developed to mitigate the adverse impact of chronic pain in everyday activities (Attridge et al., 2019). First promising results are already available that demonstrate cognitive improvement through cognitive training in patients with chronic pain (Baker et al., 2018).

## CONFLICT OF INTEREST

The authors declared that they have no conflict of interest to disclose.

## Endnote

^1^
The sample reported largely overlaps with but is not the same as the sample reported by Gubler et al. (2021) . Although all subjects in the two studies followed the same procedure, the samples differ due to different exclusion criteria. Of the 33 patients with chronic pain in this study, 20 patients were also in the chronic pain sample reported in the study of Gubler et al. (2021).
